# The Vitamin D Status of Prison Inmates

**DOI:** 10.1371/journal.pone.0090623

**Published:** 2014-03-05

**Authors:** Benjamin Udoka Nwosu, Louise Maranda, Rosalie Berry, Barbara Colocino, Carlos D. Flores Sr., Kerry Folkman, Thomas Groblewski, Patricia Ruze

**Affiliations:** 1 Division of Endocrinology, Department of Pediatrics, University of Massachusetts Medical School, Worcester, Massachusetts, United States of America; 2 Department of Quantitative Health Sciences, University of Massachusetts Medical School, Worcester, Massachusetts, United States of America; 3 Department of Correction, Massachusetts Partnership for Correctional Healthcare, Norton, Massachusetts, United States of America; 4 Department of Family and Community Medicine, University of Massachusetts Medical School, Worcester, Massachusetts, United States of America; University of Tennessee, United States of America

## Abstract

**Introduction:**

There is no comprehensive, systematic analysis of the vitamin D status of prisoners in the scientific literature.

**Objective:**

To investigate the vitamin D status and its determinants in US prison inmates.

**Hypothesis:**

Given the uniformity of dietary intake amongst inmates, vitamin D status will be determined by non-dietary factors such as skin pigmentation, security level-, and the duration of incarceration.

**Subjects and Methods:**

A retrospective study of 526 inmates (males, n = 502, age 48.6±12.5 years; females, n = 24, age 44.1±12.2) in Massachusetts prisons. Vitamin D sufficiency, insufficiency, and deficiency were respectively defined as a 25(OH)D concentration 75 nmol/L; 50 to 75 nmol/L; and <50 nmol/L. The Massachusetts Department of Correction Statement of Nutritional Adequacy stated that each inmate received the recommended daily allowance of vitamin D daily. Security level of incarceration was designated as minimum, medium, and maximum. Racial groups were categorized as Black, white, Asian, and Others.

**Results:**

Serum 25(OH)D levels peaked in summer and autumn, and decreased in winter and spring. Vitamin D deficiency occurred in 50.5% of blacks, 29.3% of whites, and 14.3% of Asian inmates (p = 0.007). Black inmates had significantly lower serum 25(OH)D level than white inmates at the maximum security level (p = 0.015), medium security level (p = 0.001), but not at the minimum security level (p = 0.40). After adjusting for covariates black inmates at a maximum security level had a four-fold higher risk for vitamin D deficiency than white inmates at the same security level (OR 3.9 [95% CI 1.3–11.7].

**Conclusions:**

The vitamin D status of prison inmates is determined by skin pigmentation, seasons, and the security level of incarceration.

## Introduction

The United States has the highest prisoner population per capita of any country in the world [1]. More than 8 million offenders are under the control of US correctional agencies, and these prisoners have poor nutritional status and poor health [2] compared to the general population [3]. Recent studies of the United Kingdom and Unites States prison inmates showing poor intake of vitamin D [4], [5] and other nutrients have prompted a call for epidemiological studies devoted to criminology [1]. This call is timely as there is no systematic analysis of the vitamin D status of prisoners in global scientific literature despite recent studies linking vitamin D deficiency to poor skeletal health, lower muscle strength, increased body sway, falls, disability, low bone mineral density, osteoporosis, and fractures [6]–[10].

The term vitamin D refers to a group of seco-steroid compounds, namely vitamin D_2_ and D_3_, which are prohormones with skeletal and extra-skeletal functions [11]. Both vitamin D_2_ and D_3_ are hydroxylated by cytochrome P450 enzymes in the liver to form 25-hydroxyvitamin D [25(OH)D] which is the primary biomarker of vitamin D status. A further hydroxylation step in the kidneys yields the biologically active form of vitamin D called 1,25-dihydroxyvitamin D or calcitriol, which serves to maintain plasma calcium and phosphate concentrations, thereby promoting bone health [12].

A comprehensive analysis of the vitamin D status of prison inmates is the first step in the understanding of the impact of hypovitaminosis D on their bone health. Such an evaluation would form the basis for interventions to address the deleterious consequences of vitamin D deficiency-related bone diseases on inmates, their families, and the national healthcare budget. Our aim was to determine the baseline vitamin D profiles of prison inmates, and to investigate the non-dietary determinants of vitamin D status in this population. We hypothesized that (1) a high proportion of our cohort would be vitamin D deficient; and (2) given the uniformity of dietary intake, vitamin D status in prison inmates would depend principally on non-dietary factors such as the degree of sun exposure, skin pigmentation, security level, and the duration of incarceration.

## Subjects and Methods

### Ethics Statement

The study protocol was jointly approved by the Massachusetts Department of Public Health Institutional Review Board, the Massachusetts Department of Correction, and the University of Massachusetts Institutional Review Board. All patient records and information were anonymized and de-identified prior to analysis.

### Subjects

**Figure 1:**
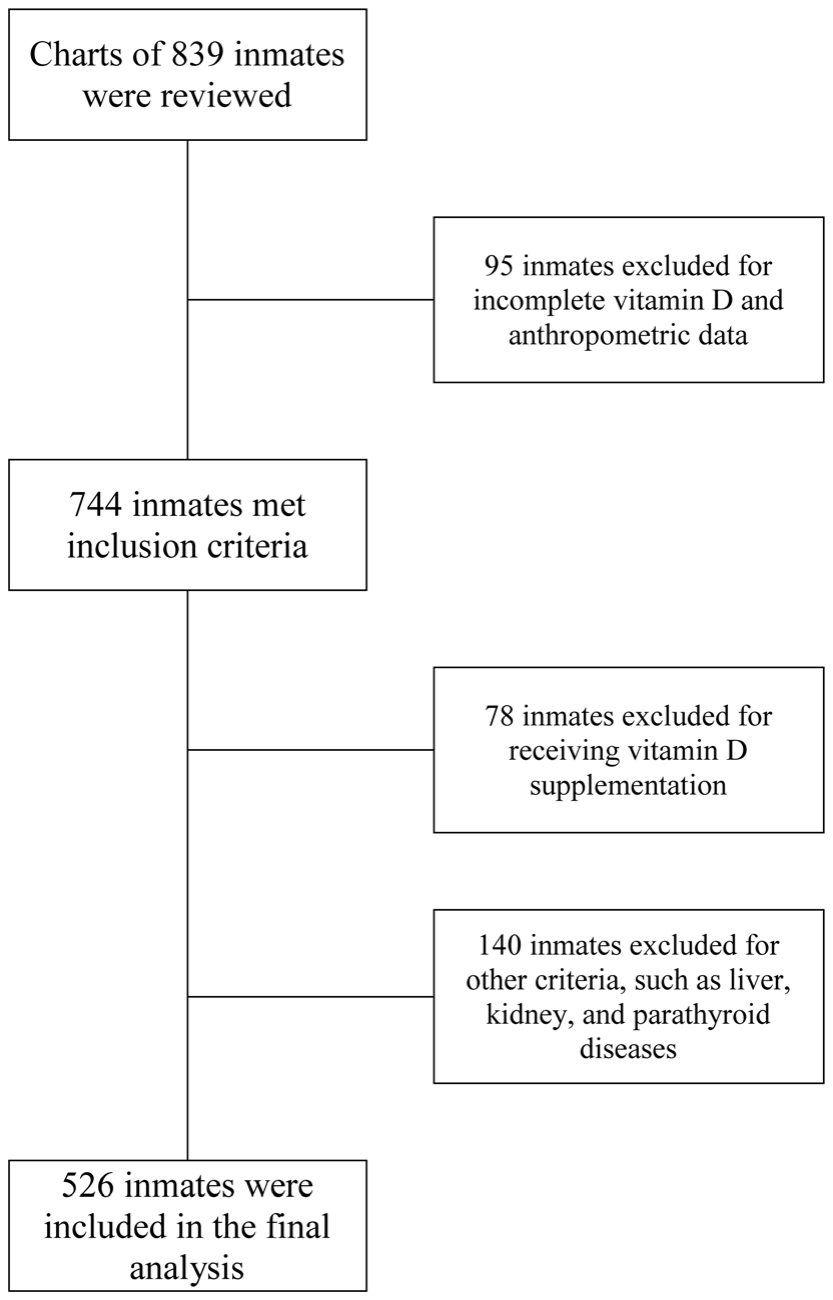
Study Flow Diagram.

All data were sourced from the Massachusetts Department of Correction's Inmate Management System (IMS) database. The medical records of 839 prison inmates in the 18 facilities in the Massachusetts Department of Correction system from June 1, 2010 through June 30, 2012 were reviewed (Figure 1). Of these, 744 inmates had complete data. Study subjects were included if they had serum 25(OH)D level obtained. The serum 25(OH)D estimations were conducted as part of routine biochemical laboratory testing in the inmates, and not due to any specific investigation for pathologies associated with vitamin D metabolism. Subjects were excluded if they were receiving pharmacological vitamin D or calcium supplementation prior to the date of 25(OH)D measurement. Seventy-eight inmates were excluded based on the above criteria. Subjects were further excluded if they were pregnant, lactating, receiving medications that affect vitamin D metabolism, or had a concurrent diagnosis of any disease that affects calcium or vitamin D metabolism such as diseases of bone, parathyroid gland, kidney, and liver. Specifically, subjects with serum creatinine of >1.5 mg/dL, or alanine aminotransferase of >2.5 times the upper limit of the normal range were excluded [13]. An additional 140 inmates were excluded based on these latter exclusion criteria. Five hundred and twenty-six (526) inmates (males, n = 502, age 48.6±12.5 years; females, n = 24, age 44.1±12.2) met the inclusion criteria and were analyzed for this study. Because vitamin D status could vary with sunlight exposure and the seasons, we categorized each inmate's date of vitamin D draw according to the seasons as follows: fall (September 22–December 21), winter (December 22–March 21), spring (March 22–June 21), and summer (June 22–September 21) [14].

### Methods

#### Vitamin D Intake

All subjects received diets that were analyzed and approved under the Statement of Nutritional Adequacy of the Massachusetts Department of Correction using the Food Processor Structured Query Language (version 10.12) Nutritional Analysis System from the Elizabeth Stewart Hands and Associates (ESHA) Research, Salem, Oregon. The Statement of Nutritional Adequacy stated that the inmates' menus met or exceeded the recommended daily allowance (RDA) for vitamin D of 600 IU, as specified by the Dietary Reference Intakes (DRI) of the Food and Nutrition Board, National Academy of Sciences, National Research Council, and the American Correctional Association [15].

#### Anthropometry

Anthropometry was measured by correctional officers. Height and weight were measured in light clothing, with no shoes, using an upright scale. Body mass index (BMI) was derived using the formula weight/height^2^ (kg/m^2^). Age of study subjects was determined by the date of the 25(OH)D measurement. Subjects were categorized into normal weight, overweight, and obese groups, based on BMI criteria as BMI <25 kg/m^2^, 25–29.9, and ≥30 kg/m^2^ respectively.

#### Assay

Serum levels of 25(OH)D were analyzed using 25-hydroxy chemiluminescent immunoassay (DiaSorin Liaison; Stillwater, Minnesota), which has a 100% cross-reactivity with both metabolites of 25(OH)D namely, 25(OH)D_2_ and 25(OH)D_3_ and thus measures total serum 25(OH)D content. Its functional sensitivity is 4 ng/mL (10 nmol/L), and its intra- and inter-assay coefficients of variation are 5% and 8.2% respectively.

#### Vitamin D Status

Vitamin D sufficiency was defined as a 25(OH)D concentration ≥75 nmol/L; vitamin D insufficiency as 25(OH)D of 50 to 75 nmol/L; and vitamin D deficiency as 25(OH)D level <50 nmol/L according to the Endocrine Society criteria [16].

## Statistical Analyses

Statistical analyses were performed using the SPSS Predictive Analytics SoftWare v.21 (IBM Corporation, Armonk, NY) and Microsoft Excel (2007). Means and standard deviations were calculated for descriptive summary statistics and 25(OH)D measurements. Anthropometrics and 25(OH)D levels were compared using Student's *t* test. Race, security level, and seasons of blood draw were compared using Fisher's exact test. A two-way ANOVA was first used to explore the differences in 25(OH)D between the four racial groups (Black, white, Asian, and Others) [F (3,522) = 7.836, p<0.001]; as well as the 3 security levels of incarceration [F (2,523) = 3.568, p = 0.029], followed by post-hoc comparisons. Data were expressed as mean ± standard deviation (SD). Linear regression statistics was used to determine the relationship between the duration of incarceration and serum 25(OH)D level. The duration of incarceration was log transformed to achieve normality.

For the analyses, subjects were first stratified by gender to determine the effect of gender on serum 25(OH)D concentrations. They were then stratified by racial groups according to the official designation of inmates by the Massachusetts Department of Correction into white, black, Asians, and Others (consisting of Native Americans, Pacific Islanders, and Alaskan natives), to determine the relationship between race and 25(OH)D levels. We further investigated the relationship between 25(OH)D level and the duration of incarceration. Finally, the inmates were stratified by their level of incarceration into minimum-, medium-, and maximum security levels to investigate the effects of levels of incarceration and sun exposure on serum 25(OH)D concentrations. With respect to sun exposure, inmates at the minimum security level were allowed an average daily sun exposure of about 5–10 hours/day during which time they may engage in exercise, working outside the facilities as road crews, park maintenance crew, or in farms. Inmates at the medium security level spent an average of 1–5 hours/day in recreational activities under supervision. Inmates at the maximum security level spent only one hour in recreational activities per day under a heavy guard.

## Results

### Patient Characteristics Stratified by Gender

Table 1 shows a comparative analysis of the inmates' characteristics stratified by gender. The male inmates were taller, and had significantly longer duration of incarceration than the female inmates. A significantly higher proportion of female inmates were incarcerated at the minimum security level compared to male inmates. In contrast, a higher proportion of male inmates were incarcerated at the medium security level. Only male inmates were incarcerated at the maximum security level.

**Table 1:** Characteristics of Prison Inmates Stratified by Gender.

Parameter	Males n = 502	Females n = 24	*p*
Age (yr)	48.6±12.5	44.1±12.2	0.09
**Height (m)**	**1.8±0.1**	**1.7±0.09**	**<0.001**
**Weight (kg)**	**90.6±19.4**	**77.2±17.7**	**0.001**
Body Mass Index (kg/m^2^)	29.1±5.4	28.1±6.0	0.40
<25	22.7±1.8 n = 104	22.0±2.0 n = 8	0.24
25–29.9	27.4±1.5 n = 215	27.4±1.7 n = 8	0.90
≥30	34.7±4.3 n = 183	34.9±4.1 n = 8	0.90
25-hydroxyvitamin D (nmol/L)	62.3±27.4	60.8±31.6	0.79
<50	33.9±9.5 n = 168	33.4±8.7 n = 9	0.91
50–75	60.5±7.0 n = 175	59.3±9.2 n = 8	0.60
≥75	94.1±18.7 n = 159	97.4±30.6 n = 7	0.66
**Duration of Incarceration (yr)**	**7.0±9.1**	**2.8±3.7**	**0.024**
Race			
White	371 (73.9%)	21(87.5%)	0.16
Black	105 (20.9%)	2 (8.3%)	0.19
Asian	7 (1.4%)	0 (0%)	1.00
Other	19 (3.8%)	1 (4.2%)	1.00
Security Level of Incarceration			
** Minimum**	**45 (9.0%)**	**19 (79.2%)**	**<0.001**
** Medium**	**388 (77.3%)**	**5 (20.8%)**	**<0.001**
Maximum	69 (13.7%)	0 (0%)	0.059
Seasons			
Summer	140 (27.9%)	5 (20.8%)	0.50
Fall	115 (22.9%)	4 (16.7%)	0.62
Winter	129 (25.7%)	6 (25.0%)	1.00
Spring	118 (23.5%)	9 (37.5%)	0.14

### Proportion of subjects with vitamin D deficiency, insufficiency, and sufficiency

Table 2 shows that the overall proportion of inmates in our cohort with vitamin D deficiency is 33%, vitamin D insufficiency 34%, and vitamin D sufficiency 31%. Vitamin D deficiency occurred in 50.5% of black inmates, 29.3% of white inmates, 14.3% of Asian inmates, and 35% of the Others (p<0.001). There was no significant difference in the proportion of inmates with vitamin D insufficiency among the four groups (p = 0.90). Vitamin D sufficiency occurred in 17.8% of black inmates, compared to 35.5% of white inmates, 42.9% of Asian inmates, and 25% of Others (p = 0.03) (Table 2).

**Table 2:** Proportion of Inmates with Vitamin D Deficiency, Insufficiency, and Sufficiency within each Racial Category.

25-hydroxyvitamin D (nmol/L)	White (n = 392)	Black (n = 107)	Asian (n = 7)	Other (n = 20)	*p*
**<50**	**(115) 29.3%**	**(54) 50.5%**	**(1) 14.3%**	**(7) 35.0%**	**0.007**
50–75	(138) 35.2%	(34) 31.8%	(3) 42.9%	(8) 40%	0.899
**≥75**	**(139) 35.5%**	**(19) 17.8%**	**(3) 42.9%**	**(5) 25.0%**	**0.030**

### The Determinants of Vitamin D status in Prison Inmates

#### Age

There was no relationship between age and 25(OH)D level in all inmates (β = −0.033, adjusted r^2^ = 0.001, p = 0.45).

#### Race

**Figure 2:**
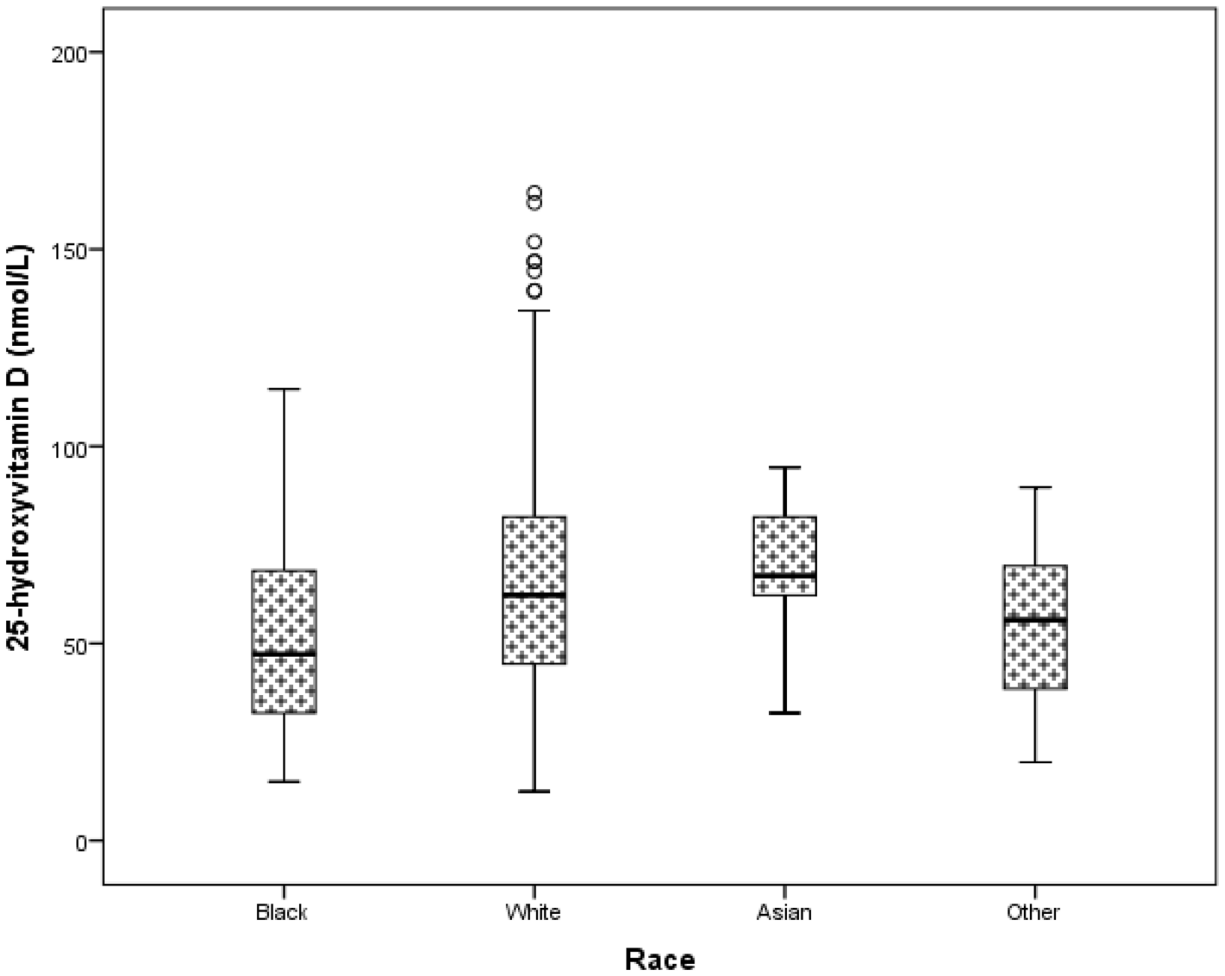
Box plots of the comparison of 25-hydroxyvitamin D values of inmates stratified by race. This figure shows that black inmates have a significantly lower 25(OH)D level compared to the white inmates (p<0.001), but similar levels to inmates categorized as Others (p = 0.54). There was inadequate power to detect a difference between black (n = 107) and Asian (n = 7) inmates (p = 0.1). Note: 50 nmol/L = 20 ng/mL.

There was a significant difference in 25(OH)D level across the racial groups (p<0.001). Black inmates had significantly lower 25(OH)D level compared to the white inmates (51.5±22.2 nmol/L vs. 65.3±28.6, p<0.001). However, blacks had similar 25(OH)D levels to the inmates categorized under Others (51.5±22.2 nmol/L vs. 55.5±.5, p = 0.54) (Figure 2). We did not have adequate power to determine a difference between blacks (n = 107) and Asians inmates (n = 7) (51.5±.2 nmol/L vs. 69.0±20.4, p = 0.10). There was no difference in the serum concentrations of 25(OH)D between the white and Asian inmates (65.3±28.6 nmol/L vs. 69.0±20.4, p = 1.0).

#### Race and Gender

When the inmates were stratified by race and gender, the black male inmates had significantly lower 25(OH)D levels than the white male inmates (p<0.001). There was, however, no significant difference in 25(OH)D levels between black and Asian male inmates (p = 0.10), nor between the black male inmates and Others (p = 0.55). Both the white and Asian male inmates had similar vitamin D levels (p = 0.73). There was equally no difference in 25(OH)D level between the black and white female inmates (p = 0.58).

#### Seasons

**Figure 3:**
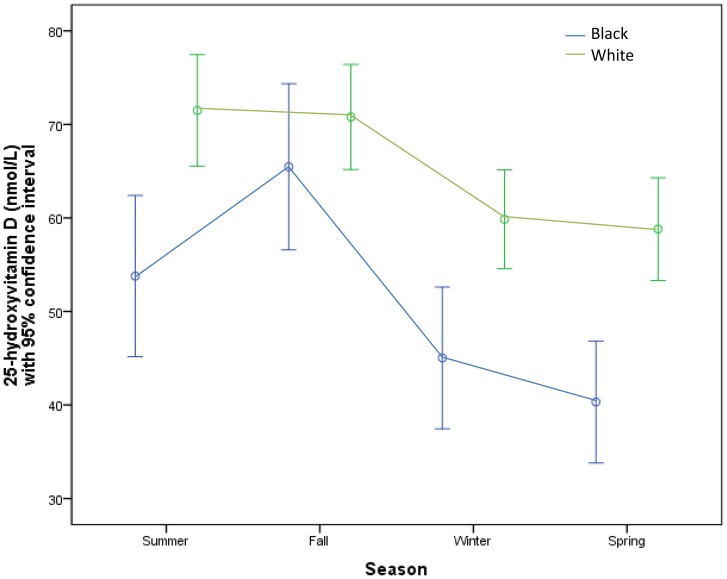
Graph of the relationship between 25(OH)D and the seasons in black and white prisoners. Black inmates had significantly lower 25(OH)D levels in all of the seasons except for Fall.

Even though serum 25(OH)D levels peaked in Summer and Fall, and reached a nadir in Winter and Spring in all racial groups, black inmates had significantly lower mean 25(OH)D levels for all seasons of the year except for Fall, compared to white inmates (Figure 3). The seasonal pattern of change in the serum concentrations of 25(OH)D with seasons in blacks vs. whites were as follows: Summer (53.7±23.1 nmol/L vs. 71.5±.4, p = 0.005), Fall (65.5±.5 nmol/L vs. 70.8±26.5, p = 0.35), Winter (45.0±18.0 nmol/L vs. 59.9±27.2, p = 0.012), Spring (40.3±16.1 nmol/L vs. 58.8±26.4, p = 0.001).

#### Duration of Incarceration

**Figure 4:**
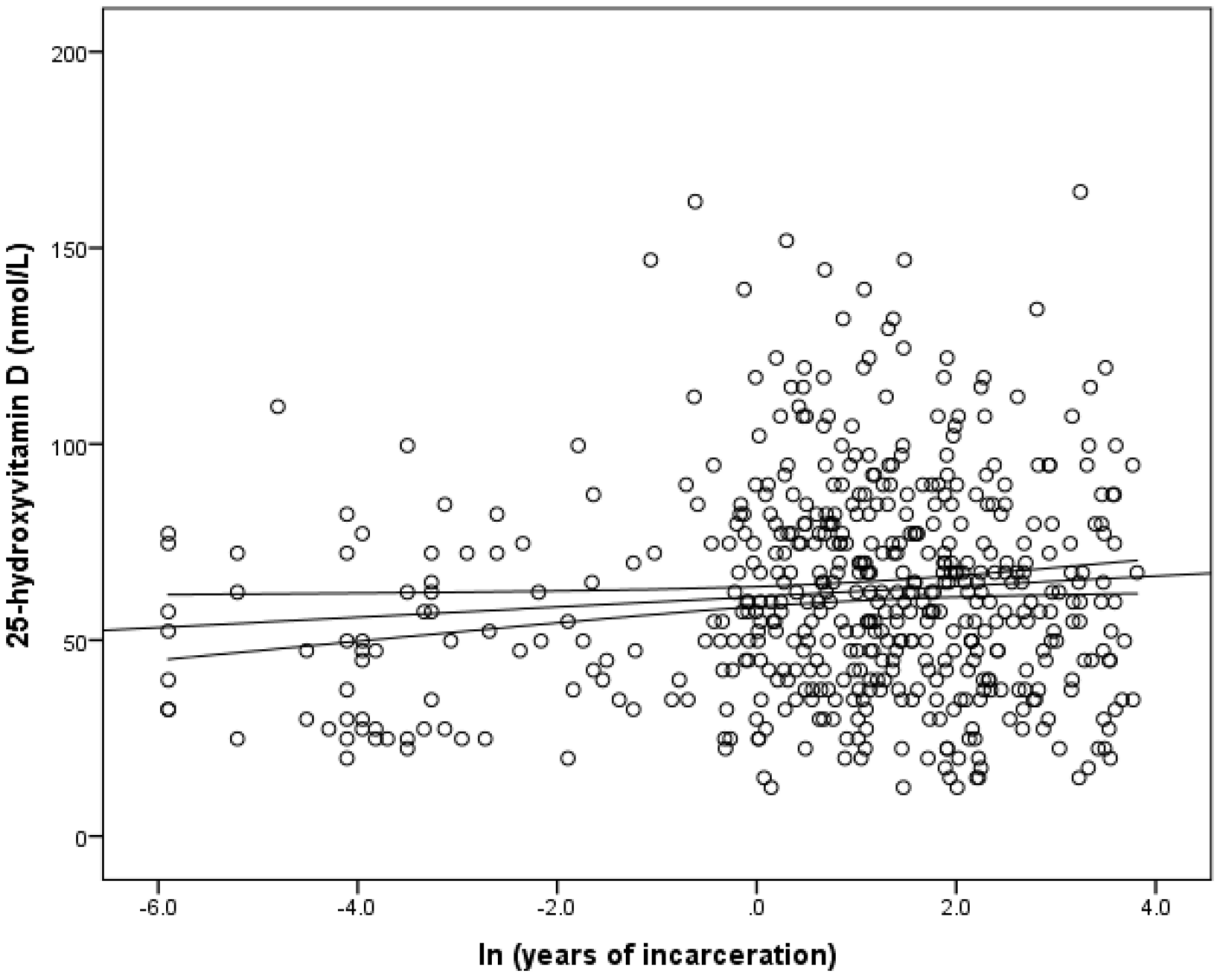
The relationship between serum concentrations of 25-hydroxyvitamin D [25(OH)D] and years of incarceration. This figure shows a weakly significant relationship between the years of incarceration and serum concentration of 25(OH)D in all inmates after adjusting for age, race, season, and BMI (β = 0.09, r^2^ = 0.07, p = 0.042). A similar investigation in inmates in the maximum security level showed no significant relationship between 25(OH)D and the duration of incarceration (β = 0.09, r^2^ = 0.09, p = 0.49).

After adjusting for age, sex, season, and BMI, there was a weakly significant relationship between the duration of incarceration and serum 25(OH)D level in all inmates (β = 0.91, adjusted r^2^ = 0.07, p = 0.042) (Figure 4). However, a further investigation of the above relationship in patients at the maximum security level showed no significant relationship (β = 0.91, adjusted r^2^ = 0.09, p = 0.49).

#### Security Level of Incarceration

**Figure 5:**
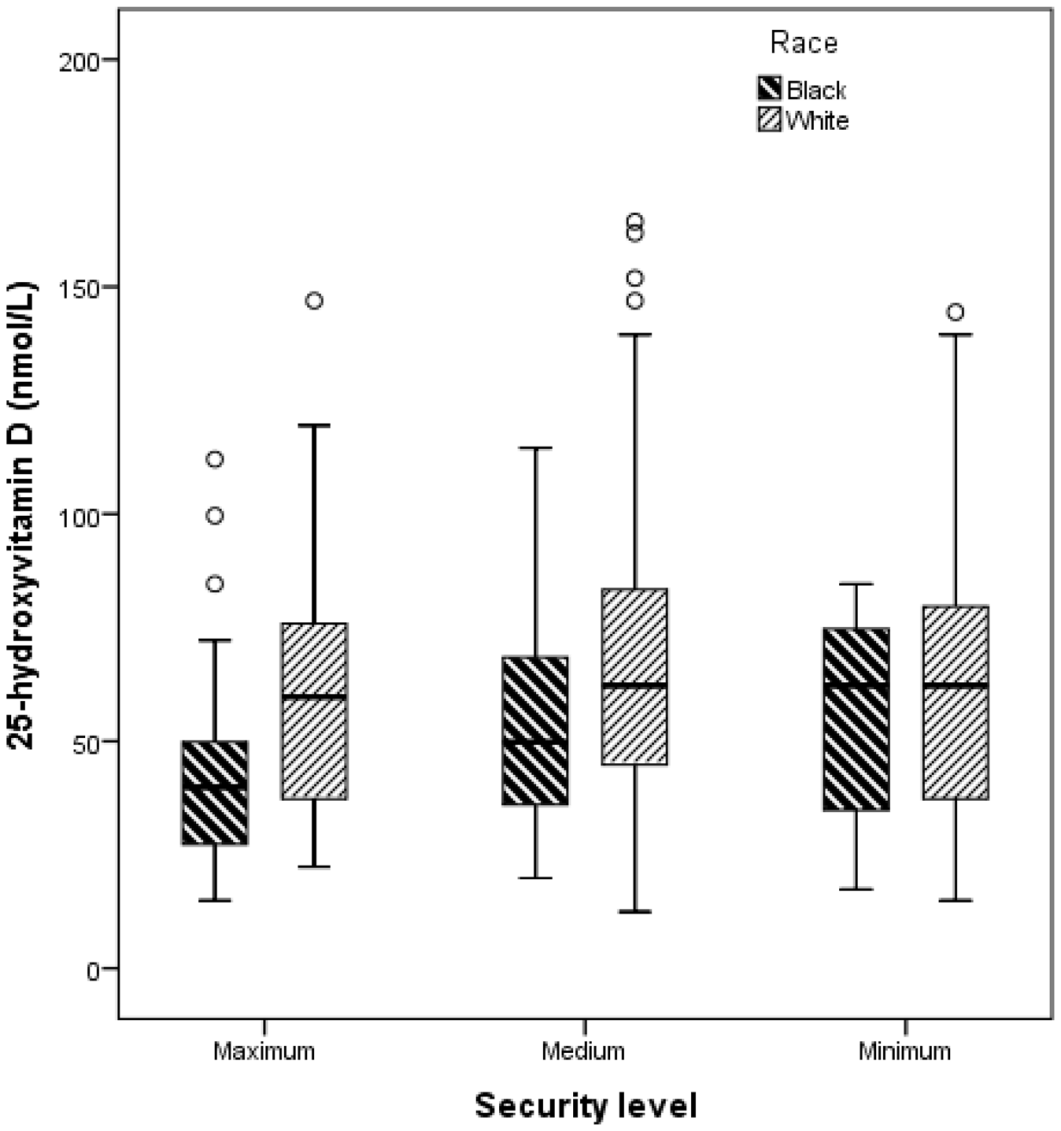
Complex box plots of the comparison of 25-hydroxyvitamin D values of inmates stratified by both race and the security level of incarceration. This figure shows a significant difference in 25(OH)D level between the black and white inmates at both the maximum security level (p = 0.015), and the medium security level (p = 0.001), but not at the minimum security level (p = 0.40). Note: 50 nmol/L = 20 ng/mL.

Inmates at the maximum security level had significantly lower 25(OH)D level (54.1±28.0 nmol/L) compared to those at the medium- (63.7±27.4 nmol/L), or the minimum security level (61.7±27.4 nmol/L) (p = 0.029). Further stratification by race showed a significant difference in 25(OH)D level between the black and white inmates at the maximum security level (44.3±24.0 nmol/L vs. 61.1±28.5, p = 0.015), and at the medium security level (53.5±20.8 nmol/L vs. 66.3±28.7, p = 0.001), but no difference at the minimum security level (55.4±24.1 nmol/L vs. 62.8±28.7, p = 0.40) (Figure 5). After adjusting for age, sex, BMI, seasons, skin tone, eye and hair color, black inmates incarcerated at a maximum security level were approximately 4 times more likely to be vitamin D deficient than white inmates at the same security level (OR 3.9 [95% CI 1.3–11.7]. Black inmates at medium security level were 2.5 times more likely to be vitamin D deficient compared to white inmates in the same security level (OR 2.5 [95% CI 1.4–4.4]. There was no difference in risk between black and white inmates at the minimum security level (OR 1.9 [0.45–7.7].

## Discussion

This study has 3 important findings: first, only 31% of prison inmates in our cohort were vitamin D sufficient, while the rest were either vitamin D deficient (33%), or vitamin D insufficient (34%). Second, a higher proportion of black inmates, regardless of their incarceration level, had lower 25(OH)D level compared to the non-black inmates. Third, after adjusting for age, sex, BMI, seasons, skin tone, eye and hair color, black inmates incarcerated at a maximum security level were 4 times more likely to be vitamin D deficient than white inmates at the same security level. Age, gender, and the duration of incarceration were not significant determinants of inmates' vitamin D status.

The Statement of Nutritional Adequacy of the Massachusetts Department of Correction states that the inmates received the RDA for vitamin D in their diet. However, given that 67% of the inmates in our cohort had hypovitaminosis D, it is most likely that the amount of vitamin D in their diet was not sufficient to maintain a normal vitamin D status especially in the colder months, or that the process of food handling in prisons might have resulted in reduced amounts of vitamin D in their diet. This is supported by studies that either documented poor intake of vitamin D by inmates as a result of subnormal vitamin D contents of foods served in correctional facilities, or suggested that the method of food handling in correctional facilities could lead to vitamin D deficiency [4], [5]. Eves et al [4] reported that even though vitamin D intakes in correctional facilities could be in excess of recommended daily allowance, extensive warm-holding of foods prior to service could result in significant losses of some vitamins. Thus, the actual amounts of vitamin D consumed may be markedly less than the initial estimates by dietary analyses [4]. This scenario may be applicable to our study. Therefore, even though our study demonstrates that the independent determinants of vitamin D status in prison population are skin pigmentation, security level of incarceration, and seasons; dietary factors such as excessive warm holding of fold before service may also lead to subnormal amounts of vitamin D in diet, and consequent hypovitaminosis D.

This is the first in-depth examination of the unique determinants of vitamin D status of prison inmates in literature. First, this study confirms the effects of both isolation and insolation on vitamin D status. Insolation is defined as the incoming solar radiation that reaches the earth, its atmosphere, and surface objects [17]. Insolation is the primary source of vitamin D [11]. This study demonstrates that individuals confined or restricted to similar environments such as prisons, where they are exposed to a similar pattern of nutritional intake, could have variable vitamin D status based on their level of insolation. Second, this study demonstrates the effect of skin pigmentation on vitamin D status in prison inmates. It shows that under conditions where individuals are exposed to a similar pattern of nutritional intake, darkly pigmented subjects would have lower serum concentrations of 25(OH)D compared to non-pigmented individuals. This is because melanin, which is found throughout the epidermis, is a natural sunscreen that absorbs ultraviolet B radiation thereby reducing the amount of vitamin D produced during sun exposure [18], [19]. Third, our study shows that age, gender, and the duration of incarceration have no biologically significant effect on inmates' vitamin D status.

These findings have implications at three concentric levels: the inmates [1], [9], [10], the correctional system, and the national healthcare policy [1], [4], [5]. At the level of the inmates, prolonged vitamin D deficiency could lead to poor health, as strong associations between vitamin D deficiency and increased risk for several diseases such as type 1 and type 2 diabetes, cardiovascular diseases, rheumatoid arthritis, infectious diseases, depression, and cancers of the breast, prostate, colon, and pancreas, have been reported [11], [20], [21]. Though there is an ongoing debate on the significance of these extra-skeletal functions of vitamin D in humans [6], [16], there is a universal consensus on its skeletal functions, as vitamin D has been demonstrated to be vital for bone mineralization, maintenance of bone strength, and the prevention of fractures and consequent immobilization [6], [22].

At the level of the correctional facilities, this study shows that a large proportion of inmates have both vitamin D insufficiency and deficiency despite dietary analyses showing intakes above the RDA [4], [5]. Therefore, to ensure optimal bone and muscle health, and prevent functional limitations in inmates, adequate vitamin D supplementation protocol [6], [16], coupled with regular monitoring of serum 25(OH)D levels occurring once or twice yearly will be necessary. The timing of the biannual vitamin D monitoring should be in the late fall and late winter seasons. In addition, inmates at the medium and maximum security levels, especially those with dark skin pigmentation, should be monitored closely, as they are at the greatest risk for vitamin D deficiency. Furthermore, the inmates in the high risk group may require a higher dose of vitamin D supplementation, of approximately 2000–4000 IU per day, to maintain 25(OH)D above 30 ng/mL which is the threshold recommended by the Endocrine Society [16]. Finally, correctional facilities or public health officials should institute periodic verification of the actual estimates of micronutrients and vitamins contained in inmates' meals by a process of direct measurement of the vitamin D content of homogenized samples of inmates' meals to ensure that the RDA is consistently met.

At the national healthcare budgetary level, the implementation of a structured supplementation protocol is critical to the preservation of inmates' bone health [22], muscle strength [9], and the prevention of comorbidities derived from vitamin D deficiency-associated skeletal and muscular defects, such as falls, fractures, and immobilization [10]. Such a supplementation protocol will help reduce the unnecessary healthcare expenditures on these preventable comorbidities.

This study has some limitations. First, the cross-sectional study design limits causal inference on the effects of seasons, race, and security level of incarceration on vitamin D status. Second, we did not study the entire Massachusetts prison population, but only those with laboratory values for 25(OH)D who met the inclusion criteria. This limited our ability to determine the prevalence of vitamin D deficiency or sufficiency in this population. We also did not have adequate power to detect a significant difference in 25(OH)D level between black and Asian male inmates because of the very few Asian male inmates in our cohort. Third, we neither administered a food-recall questionnaire, nor performed a biochemical quantification of the 25(OH)D content of the inmates' meals to accurately determine the vitamin D content of their diet. Our attempts to obtain samples of inmates' meals for quantification of the vitamin D content were unsuccessful. However, we reviewed both the Statement of Nutritional Adequacy and the daily logs of inmates' menus for the entire Massachusetts Correctional System for uniformity of RDAs for vitamin D. Fourth, we did not have data on parathyroid hormone levels which could be elevated in states of vitamin D deficiency and hypocalcemia. Fifth, our results were derived from a prison system in a single state in the United States, therefore, we are uncertain that our results are generalizable to other correctional systems, states, or countries. Finally, the study prison sites were located around latitude 42° N, thus, it is not certain whether similar results would be obtained in correctional facilities in other geographical locations.

The unique strength of this study is that it was conducted exclusively amongst inmates living in a uniform prison system with clear guidelines regarding nutrition, security level of incarceration and their associated hours of insolation, as well as the number of hours permitted for recreational activities. We had full access to the IMS database which enabled us to accurately confirm our inclusion and exclusion criteria. We had a fairly large study sample size to enable us detect differences between the groups of interest. Our sample contained a fair representation of the major racial groups, thus enabling us to analyze the effects of differential insolation on racial groups. We had a fair distribution of inmates in each of the three major security levels of incarceration, thus, enabling us to investigate the effect of security level on vitamin D status. The lack of a significant difference in 25(OH)D levels between the female inmates of different racial groups could be due to the fact that they were all housed in the single female correctional facility in Massachusetts, and none of the female inmates was being held at the maximum security level. The lack of a significant difference in 25(OH)D level between the black male inmates and the male inmates in the ‘Others’ category could be due to the fact that both groups possess variable degrees of dark skin pigmentation. Representative data on 25(OH)D levels were available for all the seasons of the year, thus allowing us to determine the variability of 25(OH)D with the seasons.

In conclusion, this study shows that in a prison system with uniform nutritional guidelines, three principal factors determine inmates' vitamin D status: skin pigmentation, seasons, and the security level of incarceration. The roles of diet and the method of food handling deserve further investigation as this study demonstrates a high occurrence of hypovitaminosis D amongst prisoners receiving the RDA of vitamin D in their diet. A comprehensive skeletal health policy that emphasizes the maintenance of normal serum 25(OH)D concentration is needed to ensure optimal skeletal and muscular health in inmates. Such a policy will ease the fiscal burden of these preventable, but costly, comorbidities on the national healthcare budget.
